# A virtual reality program to assess cognitive function in multiple sclerosis: A pilot study

**DOI:** 10.3389/fnhum.2023.1139316

**Published:** 2023-03-16

**Authors:** Wan-Yu Hsu, Joaquin A. Anguera, Albert Rizzo, Richard Campusano, Nancy D. Chiaravalloti, John DeLuca, Adam Gazzaley, Riley M. Bove

**Affiliations:** ^1^Department of Neurology, Weill Institute for Neurosciences, University of California, San Francisco, San Francisco, CA, United States; ^2^Neuroscape, University of California, San Francisco, San Francisco, CA, United States; ^3^Department of Psychiatry, University of California, San Francisco, San Francisco, CA, United States; ^4^Institute for Creative Studies, University of Southern California, Los Angeles, CA, United States; ^5^Kessler Foundation, East Hanover, NJ, United States; ^6^Department of Physical Medicine & Rehabilitation, Rutgers New Jersey Medical School, Newark, NJ, United States; ^7^Department of Physiology, University of California, San Francisco, San Francisco, CA, United States

**Keywords:** cognition, digital health, virtual reality, multiple sclerosis, cognitive assessment

## Abstract

**Introduction:** Cognitive impairment is a debilitating symptom in people with multiple sclerosis (MS). Most of the neuropsychological tasks have little resemblance to everyday life. There is a need for ecologically valid tools for assessing cognition in real-life functional contexts in MS. One potential solution would involve the use of virtual reality (VR) to exert finer control over the task presentation environment; however, VR studies in the MS population are scarce.

**Objectives:** To explore the utility and feasibility of a VR program for cognitive assessment in MS.

**Methods:** A VR classroom embedded with a continuous performance task (CPT) was assessed in 10 non-MS adults and 10 people with MS with low cognitive functioning. Participants performed the CPT with distractors (i.e., WD) and without distractors (i.e., ND). The Symbol Digit Modalities Test (SDMT), California Verbal Learning Test—II (CVLT-II), and a feedback survey on the VR program was administered.

**Results:** People with MS exhibited greater reaction time variability (RTV) compared to non-MS participants, and greater RTV in both WD and ND conditions was associated with lower SDMT.

**Conclusions:** VR tools warrant further research to determine their value as an ecologically valid platform for assessing cognition and everyday functioning in people with MS.

## Introduction

Cognitive impairment is one of the core manifestations of multiple sclerosis (MS), and it occurs in up to two-thirds of people with MS, affecting their employment, independence, and quality of life (Benedict et al., 2020). Currently, clinical cognitive assessment in MS relies on neuropsychological examination. However, most of the administered neuropsychological tasks have little resemblance to everyday life. Taking advantage of recent advances in information technology, virtual reality (VR) programs provide an innovative platform for creating a three-dimensional, dynamic environments that simulate the real world, allowing users to naturally interact with objects or to complete relevant tasks. By integrating VR simulations with cognitive tasks, this platform can provide standard and replicable task demands that allow for realistic “real-world” cognitive assessments (Parsons, 2015), and some neuropsychological tasks have been previously adapted to a VR platform (Parsons and Courtney, 2014; Stokes et al., 2022). Clinical research using VR has been successfully implemented for cognitive assessment in brain injury (Denmark et al., 2019), attention deficit hyperactivity disorder (Adams et al., 2009) and mild cognitive impairment (Wang et al., 2020). However, to date, studies using VR programs to assess cognition in people with MS are scarce (Lamargue-Hamel et al., 2015; Realdon et al., 2019).

The present study investigated the utility and feasibility of a specific VR platform to assess cognition in people with MS. A VR classroom [Virtual Reality Attention Tracker (VRAT)] embedded with a 13-min continuous performance task (CPT; Friedman et al., 1978; Michael et al., 1981) was assessed in 10 non-MS adults and 10 adults with MS with low cognitive functioning (the Symbol Digit Modalities Test (SDMT) *z*-score <−1.0 (*z*-score derived from published norms (Kiely et al., 2014))). We hypothesized that participants with MS would show lower performance on the VRAT compared to those without MS. Furthermore, we hypothesized that performance on the VRAT program would show associations with performance on standard cognitive measures [i.e., SDMT and California Verbal Learning Test—II (CVLT-II)].

## Material and methods

### Participants

Ten adults with a diagnosis of MS by 2010 Revised McDonald criteria (Polman et al., 2011) were recruited from the University of California, San Francisco Multiple Sclerosis and Neuroinflammation Center between September 2021 and September 2022. The inclusion criteria were: Expanded Disability Status Scale (EDSS; Kurtzke, 1983) no greater than 6.5, no paresis of the upper limbs, a minimum of 3 months since the last relapse, and no relapses or changes in symptomatic medications in the past 2 months, SDMT *z*-score < −1.0 based on Kiely et al. (2014). Differing from the commonly used cut-off of *z*-score < −1.5 (Amato et al., 2018), an SDMT *z*-score cut-off of −1.0 was chosen to allow for a broader range of cognitive functioning in the recruited MS participants in this pilot study, referred to as “low cognitive functioning”. The exclusion criteria were visual, auditory, and motor impairment that would reduce the ability to operate the VR program (i.e., unable to hear or see the distractors, or to push the trigger on the VR controller). A group of 10 non-MS, sex-, age-, and education level-matched adults with no chronic autoimmune diseases were also recruited, including from the UCSF staff, patient family members, and other eligible and willing volunteers. All study procedures were approved and in accordance with the ethical standards of the Committee for Human Research at the University of California, San Francisco (IRB No. 21-34026). Written informed consent was obtained from all participants.

### Procedures

The 1.5-h study visit began with standard measures (SDMT and CVLT-II), followed by the VR cognitive assessment (VRAT (*vide infra*)) and the feedback survey. Participants performed the VRAT testing under two conditions: with distractors (WD) and with no distractors (ND). The order of the two conditions was counterbalanced across participants. The study session did not include a pre-determined break, but participants were informed at the beginning of the visit that they could take a break at any time if needed.

### Symbol digit modalities test (SDMT)

SDMT is a widely used measure of information processing speed and selective attention in MS (Benedict et al., 2017) which requires the participant to substitute geometric symbols for numbers while scanning a response key. The written version of SDMT was administered. Correct responses that were made within 90 s were counted as the SDMT score.

### California verbal learning test—II (CVLT-II)

The CVLT-II is a reliable and valid measure of verbal learning and memory in MS (Gromisch et al., 2013). The total correct recall score (sum of the five trials) was used as the immediate free recall outcome measure, as it is reported to be one of the most sensitive CVLT-II measures in MS (Stegen et al., 2010).

### Virtual reality attention tracker (VRAT)

**Figure 1:**
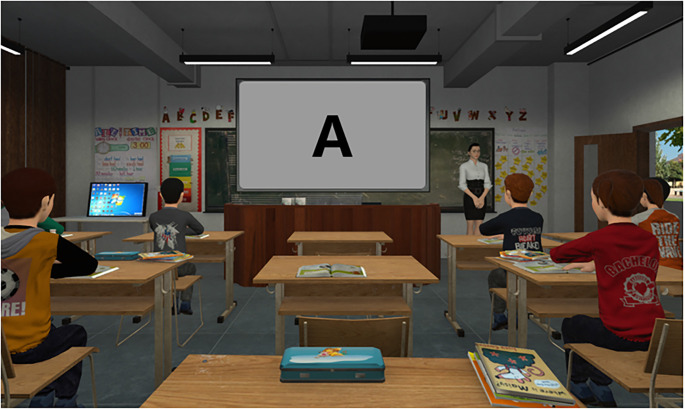
Screenshot of Virtual Reality Attention Tracker (VRAT) program.

The VRAT program (VRAT, Version 1.9, Cognitive Leap Inc.) provides a simulation of a standard classroom scenario (Rizzo et al., 2006) embedded with CPT task (Figure 1). The environment consists of the interior of a standard classroom with several student avatars, posters on the wall, desks and chairs, books on the desks, a teacher and a whiteboard in the front, two doors on the right-side wall, and windows looking out onto the street on the left-side wall. The VR environment was presented in an HTC Vive system head-mounted display (1,440 × 1,600 pixels per eye resolution, 110° field of view) connected to a desktop computer: Intel Core i7-4820K, 32 GB RAM, Windows 10. All the participants were naïve users of VR technology. However, the participants did not have any problems when adjusting to or using the HTC Vive headset, and no testing session had to be interrupted or halted due to simulator sickness.

In the VR classroom CPT assessment, the participants were required to monitor a series of letters and respond when a defined letter sequence appears. Participants were instructed by a virtual teacher to view a series of letters presented on the whiteboard. Participants had to press the trigger button on the HTC Vive controller as quickly and as accurately as they could when they saw letter “X” appear after letter “A”. Sustained (i.e., monitor a series of letters) and selective (i.e., respond when a defined letter sequence appears) attention were required to perform the task. Before starting the CPT, a 32-second practice session consists of 20 trials (including one “AX” target) without distractors was administered. The 13-min CPT task contains 520 letter stimuli (including 10% of targets “AX”) with a stimulus duration of 500 ms and an inter-stimulus interval of 1,500 ms. The CPT was administered in two conditions (13-min each): with naturalistic audio, visual, or mixed audiovisual distractors (i.e., WD: with distractors; see Table 1 for examples of distractors) and without distractors (i.e., ND: no distractors). The order of the two conditions was counterbalanced across participants. The 52 targets “AX” were equally distributed in the 13-min task, with 26 targets in the first half of the test and 26 targets in the second half of the task. A total of 30 distracters were included in the 13-min task.

**Table 1:** Examples of distractions.

Type of distractor	Example of distraction
Visual distractor	‐ Two variations of a student yawning from the desk, one of the variations is a small yawn and the other is a yawn with student leaning back stretching in chair ‐ Principal walks by the classroom door outside ‐ Student drinks from water bottle
Audio distractor	‐ Bus accelerating/driving outside ‐ Car horn ‐ A sneeze ‐ A ball bouncing outside
Audiovisual distractor	‐ A student turns around and asks another student “May I borrow a pencil please” ‐ Two variations of students walking by the classroom outside talking to each other ‐ A student turns and asks another student “May I borrow a ruler?”

### VR feedback survey

Three questions were included in the survey: “Did you enjoy the experience with the VRAT system?” (all participants), “Do you think the system will be helpful for assessment of your cognition?” (participants with MS), and “Do you think you would be able to tolerate a VR session for an hour?” (participants with MS). There were five response options for the first two questions: not at all, very slightly, slightly, much, and very much. Response options to the last question were “yes” or “no”.

### Data analysis

Variables of interest included traditional CPT measurements such as correct response rate (CR), omission errors (OE), commission errors (CE), and reaction time variability (RTV). RTV was chosen over the averaged RT because studies have reported that RTV shows a stronger association than RT with white matter integrity (Fjell et al., 2011; Tamnes et al., 2012), a measure that is highly relevant for cognitive changes in people with MS (Hulst et al., 2013). Moreover, studies have found that intraindividual variability is more sensitive to identify cognitive deficits in aging (Bielak et al., 2010) and clinical populations (Klein et al., 2006; de Frias et al., 2007) rather than mean RT. To compare these variables between participants with and without MS, student t-tests were performed. The effect size was calculated using eta square for all of the analyses. All the measured data are presented as mean ± standard error of the mean (SEM). The association between standard neuropsychological measures and VRAT performance was examined using Pearson’s correlation. The statistical analyses were performed using IBM SPSS Statistics version 22.0 (IBMs Corp). The threshold of statistical significance was set at *p* ≤ 0.05.

**Table 2:** Baseline demographic and clinical characteristics of participants enrolled in the study.

	MS (*n* = 10)	Non-MS (*n* = 10)
Age (years)	42.0 (1.3)	36.3 (2.5)
Sex	10F	8F, 2M
Education (years)	17.0 (0.4)	17.6 (1.0)
Right-handedness, n (%)	10 (100%)	10 (100%)
Part- or full-time employed, n (%)	8 (80%)	8 (80%)
Baseline SDMT score	39.3 (1.7)	56.6 (2.9)^*^
Baseline SDMT *z*-score	−1.54 (0.13)	0.07 (0.29)^*^
CVLT-II Total Correct	49.9 (2.4)	63.1 (2.9)^*^
EDSS (median ± IQR)	3 ± 1	–
Disease Duration (years)	10.5 (1.9)	–
Race, n (%)		
White	5 (50%)	7 (70%)
Black/African American	–	–
Asian	1 (10%)	2 (20%)
Other/Unknown	4 (40%)	1 (10%)
MS subtype, n (%)		
Relapsing-Remitting	10 (100%)	–
Primary Progressive	–	–
Secondary Progressive	–	–
Current DMT, n (%)		
Oral	1 (10%)	–
Self-injectable	–	–
Infused	9 (90%)	–

Abbreviations: F, female; M, male; IQR, interquartile range; SDMT, Symbol Digit Modalities Test; CVLT-II, California Verbal Learning Test-II; EDSS, Expanded Disability Status Scale; DMT, disease-modifying therapy. *p < 0.05 for the comparison between MS and non-MS groups.

## Results

### Demographic and clinical characteristics

The participants with and without MS did not differ in terms of age (*t*_13.3_ = −1.99, *p* = 0.07, *η*^2^ = 0.17) or years of education (*t*_18_ = 0.54, *p* = 0.59, *η*^2^ = 0.01; Table 2). By definition, participants with MS had lower SDMT score (*t*_18_ = 5.07, *p* < 0.001, *η*^2^ = 0.58), SDMT *z*-score (*t*_12.7_ = 5.04, *p* < 0.001, *η*^2^ = 0.58) and CVLT-II total correct number (*t*_18_ = 3.41, *p* = 0.03, *η*^2^ = 0.39) compared to those without MS.

### VRAT performance

**Figure 2:**
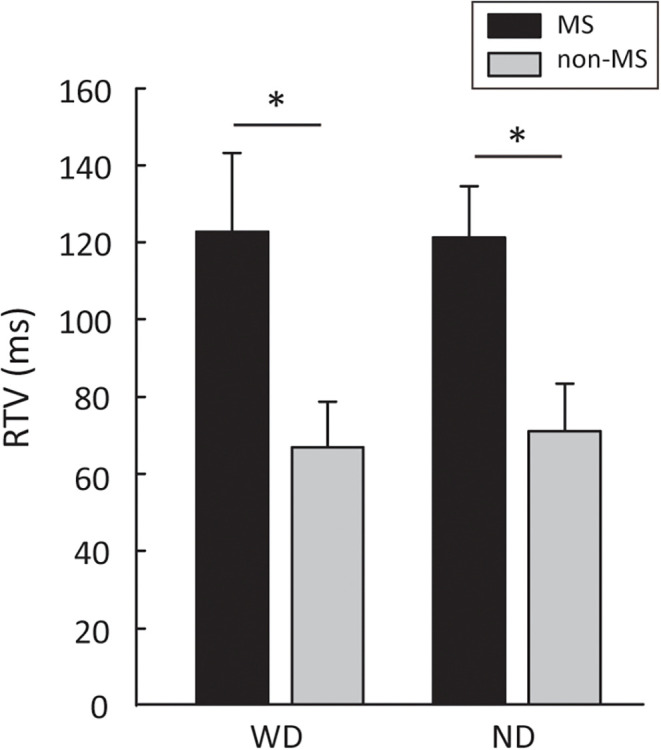
Group differences between people with MS and non-MS participants in RTV. Error bars represent SEM. MS, multiple sclerosis; RTV, response time variability; WD, with distractors; ND, no distractors. *p < 0.05.

Participants with MS showed a significantly higher RTV than participants without MS, in both the “With Distractor” (WD; 122.60 ± 20.67 ms vs. 66.87 ± 11.91 ms, *t*_18_ = −2.33, *p* = 0.03, *η*^2^ = 0.23) and “No Distractor” (ND, 121.24 ± 13.43 ms vs. 71.05 ± 12.22 ms, *t*_18_ = −2.76, *p* = 0.01, *η*^2^ = 0.29) conditions (Figure 2). In order to examine whether the difference in RTV between the two groups is more pronounced in one of the conditions (i.e., WD vs. ND), 2-way repeated measures ANOVA with group (MS, non-MS) as between-subject factor and condition (WD, ND) as within-subject factor was performed. The results showed no significant group × condition interaction (*F*_(1,18)_ = 0.13, *p* = 0.72, *η*^2^ = 0.007), suggesting that the levels of difference in RTV between the two groups are the same across the two conditions (i.e., with and without distractors).

No significant differences between the two groups in terms of CR (WD: 0.97 ± 0.01 vs. 0.98 ± 0.01, *t*_18_ = 0.45, *p* = 0.65, *η*^2^ = 0.01; ND: 0.93 ± 0.02 vs. 0.97 ± 0.01, *t*_14.2_ = 1.35, *p* = 0.19, *η*^2^ = 0.09), OE (WD: 1.1 ± 0.4 vs. 0.8 ± 0.4, *t*_18_ = 0.45, *p* = 0.65, *η*^2^ = 0.01; ND: 3.2 ± 1.1 vs. 1.4 ± 0.6, *t*_14.2_ = 1.35, *p* = 0.19, *η*^2^ = 0.09), and CE (WD: 1.6 ± 0.5 vs. 1.2 ± 0.5, *t*_18_ = −0.49, *p* = 0.65, *η*^2^ = 0.01; ND: 1.4 ± 0.4 vs. 0.6 ± 0.2, *t*_14.1_ = −1.46, *p* = 0.16, *η*^2^ = 0.10) were found.

Since the CPT task duration is long (i.e., 13 min for each condition), it is possible that task performance may decline over time due to fatigue, limited sustained attention, or other factors. To examine whether VRAT task performance changed over the 13-min task, the CPT task performance data from the first and the second half of the task were submitted to 2-way repeated measures ANOVA with group (MS, non-MS) as between-subject factor and block (first, second) as within-subject factor. The results showed no significant group × block interaction for CR (WD: *F*_(1,18)_ = 2.14, *p* = 0.16, *η*^2^ = 0.10; ND: *F*_(1,18)_ = 0.26, *p* = 0.61, *η*^2^ = 0.01), OE (WD: *F*_(1,18)_ = 2.14, *p* = 0.16, *η*^2^ = 0.10; ND: *F*_(1,18)_ = 0.26, *p* = 0.61, *η*^2^ = 0.01), CE (WD: *F*_(1,18)_ = 0.00, *p* = 1.00, *η*^2^ = 0.00; ND: *F*_(1,18)_ = 0.22, *p* = 0.64, *η*^2^ = 0.01) and RTV (WD: *F*_(1,18)_ = 0.15, *p* = 0.70, *η*^2^ = 0.008; ND: *F*_(1,18)_ = 0.54, *p* = 0.47, *η*^2^ = 0.02). Given that participants with MS had a significantly higher RTV than non-MS participants, *post-hoc* analyses were performed to confirm that the group differences can be observed in both the first and second half of the task. As anticipated, the group differences in RTV were shown in both the first half (WD: *t*_13.2_ = 2.54, *p* = 0.02, *η*^2^ = 0.26; ND: *t*_18_ = 1.86, *p* = 0.07, *η*^2^ = 0.16) and the second half (WD: *t*_18_ = 2.59, *p* = 0.01, *η*^2^ = 0.27; ND: *t*_14.1_ = 2.57, *p* = 0.02, *η*^2^ = 0.26) of the task. These results suggest that group differences in task performance were stable over the 13-min task and could be observed during the first half of the task alone.

### Association between VRAT performance and standard cognitive measures

**Figure 3:**
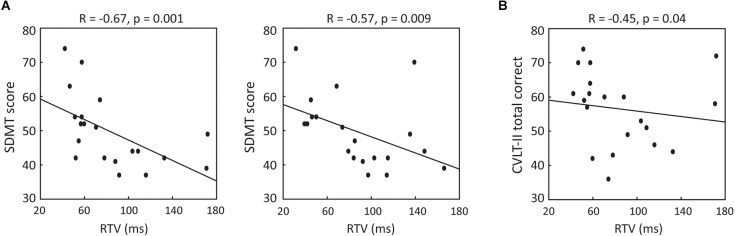
**(A)** Association between SDMT score and RTV in WD (left panel) and ND (right panel) conditions. **(B)** Association between CVLT-II total recall score and RTV in WD condition. WD, with distractors; ND, no distractors; SDMT, Symbol Digit Modalities Test; CVLT, California Verbal Learning Test-II.

To discern whether performance in the VRAT was associated with standard measures of cognition, Pearson’s correlation analyses were performed. Negative correlations between SDMT and RTV were revealed for both the WD (*r* = −0.67, *p* = 0.001) and ND (*r* = −0.57, *p* = 0.009) conditions (Figure 3A). Restricting the analyses to only participants with MS and controlling for disease duration and cognitive fatigue [measured by Modified Fatigue Impact Scale (Kos et al., 2005) cognitive subscale], resulted in stronger associations (WD: *r* = −0.82, *p* = 0.01; ND: *r* = −0.71, *p* = 0.04). Further, a negative correlation was also noted between CVLT-II total correct number and RTV in the WD (*r* = −0.45, *p* = 0.04) condition (Figure 3B), but this did not persist when restricting the analysis to participants with MS and adjusting for disease duration and cognitive fatigue as covariates. These results indicate that VRAT performance is associated with standard MS cognitive measures (i.e., SDMT performance).

### Feasibility and tolerability

The feasibility and tolerability of the VRAT program was assessed by a feedback survey administered at the end of the study visit. Sixty-five percent of participants (13 out of 20, including eight MS) reported that they enjoyed the experience with the VRAT system “much” (*n* = 8) or “very much” (*n* = 5). Seventy percent (7 out of 10) of the participants with MS agreed “much” (*n* = 3) or “very much” (*n* = 4) that the VRAT program could be helpful for assessing their cognition. Informing future development of the VR program as a cognitive assessment for people with MS, eighty percent (8 out of 10) of people with MS reported they felt they would be able to tolerate the VR session for an hour. These findings suggest acceptance by people with MS of the VR program as a tool for assessing cognition.

## Discussion

In this pilot study, a VR-based program, the VRAT, was explored for its feasibility and utility as a tool to assess cognition in people with MS. The findings suggest that VRAT can reveal group-level differences between people with and without MS, as demonstrated by a higher RTV in people with MS compared to non-MS participants. Participants with better performance on standard cognitive measures performed better on the VRAT assessment. Moreover, the participant feedback survey suggests that VRAT is feasible and tolerable as a VR-based cognitive assessment in people with MS.

In the VRAT classroom scenario, the CPT task measuring sustained (i.e., monitor a series of letters) and selective (i.e., respond when a defined letter sequence appears) attention was performed. In line with previous studies (Arnett and Strober, 2011), the results show that people with MS and low cognitive functioning are less consistent in their performance on tasks of attention/processing speed, as indicated by a higher RTV compared to non-MS participants. Intra-individual performance variability may reflect the instability of endogenous factors, such as central nervous system integrity (Hultsch et al., 2000). More variable task performance from one moment to another has been linked to fluctuation in connectivity of neuronal pathways (Kelly et al., 2008) and cognitive functioning (MacDonald et al., 2006). RTV measures aspects of cognitive functioning related to a person’s ability to consistently focus and purposefully sustain the mental effort. Studies have reported that RTV is associated with neurological conditions (Hultsch et al., 2000) and it is linked to brain networks (MacDonald et al., 2009) and white matter integrity (Tamnes et al., 2012). In MS, studies have found that increased RTV is associated with cognitive fatigue (Bruce et al., 2010; Riegler et al., 2022). Research on MS fatigue has suggested that the inflammation in cortico-striato-thalamo-cortical circuit may involve in MS fatigue (Chalah et al., 2015). Demyelination and neurodegeneration in these brain regions may also increase variability in cognitive performance and cause cognitive fatigue.

It should be noted that there were no differences between the two study groups in CE, OE, and CR. The fact that the two groups showed a similar level of correct/error rate but a different RTV, suggests that VRAT testing may be more sensitive to detecting impaired information processing speed rather than attentional impairment in people with MS. Not with standing, this notion should be taken with caution since the altered RTV is indicative of changes in sustained attention abilities (Ziegler et al., 2019).

Few studies have examined the correlation between the VR-based cognitive assessment and standard neuropsychological examination in MS (Realdon et al., 2019). The current findings showed that participants with lower RTV in VRAT showed higher scores in SDMT and CVLT-II. Of note, since the SDMT *z*-score was used as an inclusion criterion for participants with MS, the association between SDMT and VRAT may have been underestimated. Future work is needed with larger samples to understand the association between VRAT performance and standard cognitive measures.

Although preliminary, the VRAT feedback survey results suggested high patient enthusiasm for VR-based cognitive assessment. Eighty percent and 70% of enrolled people with MS reported that they enjoyed VRAT and found it helpful for cognitive assessment, respectively. These results provide the preliminary support the notion that VR-based cognitive assessments could represent a meaningful and helpful tool for detecting cognitive changes in people with MS. There are some limitations to the present study. First, the overall low sample size makes it difficult to draw a definitive conclusion with respect to the VR testing validity in people with MS. As such, the results must be interpreted with caution. Second, with an inter-stimulus interval of 1,500 ms and 10% of targets, the VRAT is probably not as challenging as tasks in daily living (e.g., medication management and bill payment tasks) that people with MS would face. Future development of VR-based cognitive assessment for people with MS should focus on designing tasks that are more relevant to everyday life functioning and making the task look like what it is supposed to measure.

Taken together, the present study provides preliminary evidence suggesting that the VRAT, a VR-based cognitive assessment, allowing for the recording and standard measurements of task performance, may be sensitive for detecting cognitive impairment and evaluating relevant everyday functioning in people with MS. Further studies with a larger cohort of people with MS are warranted to replicate current findings and to determine an ideal duration for the testing session to make it more feasible. It is also important to design tasks that are presentative of what people with MS experience in their daily living and includes real-life task characteristics (i.e., calendar planning, cooking, shopping) to enhance the ecological validity of the VR platform in cognitive assessment for MS. Success in this area could lead to future test development that could serve to enhance the ecological validity of the VR platform to better meet the needs for more reliable, valid, and *relevant* in cognitive assessment of people having MS.

## Data availability statement

The raw data supporting the conclusions of this article will be made available by the authors, without undue reservation.

## Ethics statement

The studies involving human participants were reviewed and approved by The Committee for Human Research at the University of California, San Francisco (IRB No. 21-34026). The patients/participants provided their written informed consent to participate in this study.

## Author contributions

W-YH: conceptualization, methodology, data analysis, visualization, and manuscript writing. JA, AR, NC, JD, AG, and RB: conceptualization, methodology, and manuscript editing. RC: data curation, content curation, and manuscript editing. All authors contributed to the article and approved the submitted version.
